# A Method to Measure Myeloarchitecture of the Murine Cerebral Cortex *in vivo* and *ex vivo* by Intrinsic Third-Harmonic Generation

**DOI:** 10.3389/fnana.2019.00065

**Published:** 2019-06-26

**Authors:** Michael J. Redlich, Hyungsik Lim

**Affiliations:** Department of Physics and Astronomy, Hunter College and the Graduate Center of the City University of New York, New York, NY, United States

**Keywords:** myelin, third-harmonic generation, cerebral cortex, brain, label-free imaging, nonlinear optical microscopy

## Abstract

A new label-free method is presented for measuring myeloarchitecture of the murine cerebral cortex *in vivo* and *ex vivo*. Growing evidence suggests that cortical myelination plays significant roles in neuronal plasticity and pathologies, such as multiple sclerosis (MS), but illuminating the mechanism requires longitudinal imaging of the same brains. Here we demonstrate imaging unlabeled myelinated fibers in a live mouse brain by third-harmonic generation (THG). Contrary to other label-free microscopies based on reflectance, fibers of all orientations could be visualized, i.e., radial and tangential to the pia, which is suitable for revealing the three-dimensional connectivity. The depth of THG imaging in an intact brain was approximately 200 μm, so the network of myelinated fibers could be captured into layers 2/3 *in vivo*. THG provides a novel base for reconstruction of morphology. Semi-automatic tracing of THG-positive axons unraveled the depth-dependent distribution of the myelin lattice. Finally, a unique light property of THG was exploited for the estimation of the g-ratio. The demonstrated THG morphometry of the length density, orientation, and sheath thickness of cortical myelin could be useful for elucidating its function and how it is modulated during learning and disease.

## Introduction

Although myelination in the brain is typically associated with the white matter, it is abundant also in the gray matter. Conceivably, myelin is an indispensable element for building a cortical circuit but the mechanism underlying the recruitment of cortical myelin is unknown. There can be multi-faceted functions, i.e., the thalamocortical axons myelinate to synchronize the arrival of excitation across the cortex (Salami et al., 2003), whereas another distinct function might be carried out by ensheathing the inhibitory neurons in layers 2/3 and 4 (Micheva et al., 2016). Furthermore, myelination of the neocortical pyramidal neurons has been proposed as a substrate for neural plasticity (Tomassy et al., 2014). Demyelination in the gray matter may have profound cognitive and behavioral consequences, as implicated in disorders such as multiple sclerosis (MS; Peterson et al., 2001; Bø et al., 2003; Calabrese et al., 2010). However, understanding the regulation of cortical myelin in health and pathology is hampered by the lack of suitable technology for imaging the architecture in live animals. The standard procedure to fix, embed, slice the sample, and then image the sections by electron microscopy (EM) or immunohistochemistry (IHC) is not only labor-intensive but also erases the dynamics present in fresh tissue. For monitoring experience- and age-dependent remodeling of oligodendrocytes (OLs; Hill et al., 2018; Hughes et al., 2018) without sample-to-sample variabilities, it is desirable to image the same brains longitudinally. An ability to visualize the responses to various experimental and environmental stimuli would be crucial for elucidating the principle of cortical myelination.

We demonstrate label-free imaging of cortical myelin in the mouse brain *in vivo* and *ex vivo*. Third-harmonic generation (THG), arising from index mismatch between lipid membranes and aqueous cytoplasm (Débarre et al., 2006), has been employed for visualizing myelinated fibers in the central (CNS; Farrar et al., 2011) and peripheral nervous systems (PNS; Lim et al., 2014). Here the imaging contrast was tested specifically for measuring gray matter myelinated axons in the brain, which are much thinner than those in the white matter tracts.

## Materials and Methods

### Animals

All mice were obtained from Jackson Lab, including Thy1-yellow fluorescent protein (YFP; #003709) and 2′,3′-cyclic nucleotide 3′ phosphodiesterase (CNP)-EGFP (#026105). All procedures were approved by the Hunter College Institutional Animal Care and Use Committee (IACUC). For *in vivo* imaging, a craniotomy was performed to place an optical cranial window (Holtmaat et al., 2009). Briefly, animals were anesthetized by isoflurane inhalation and placed on a temperature-controlled heating pad. The head was shaved and the scalp was cut away. A small area of the skull was removed while leaving the dura intact and a coverslip glass was attached. A head bar was cemented on the skull in order to reduce motion artifacts. After imaging, the animal was euthanized. For *ex vivo* imaging, transcardial perfusion fixation was performed with 4% paraformaldehyde. The fixed brain was sliced using a vibratome (Leica VT1200S) and then transferred to a dish and held down with an anchor.

### THG and Two-Photon Fluorescence (2PEF) Microscopy

For THG microscopy (THGM), a standard setup was employed as described previously (Lim et al., 2014). For the excitation of THG, short pulses from an optical parametric oscillator (OPO) pumped with 100-fs, 80-MHz repetition rate Ti:Sapphire laser were used (Chameleon; Coherent, Inc.). The excitation wavelength was 1,160 nm. The elliptical polarization was obtained with half- and quarter-waveplates. The excitation beam was focused with a water-dipping objective lens (Nikon CFI75 16 × 0.8 NA or Leica HC FLUOTAR L 25 × 0.95 NA). The average power was approximately 100 mW at the sample. The backward-scattered THG signal from the brain was collected with the same objective lens and detected with a photomultiplier tube (PMT; Hamamatsu H10770PA-40). The pixel dwell time was ~3 μs. Typically 1–5 frames were acquired at the frame rate of ~1.5 Hz. For simultaneous acquisition of two-photon excitation fluorescence (2PEF) and THG, a beam of short pulses at 850 nm from an independent mode-locked Ti:Sapphire laser (Tsunami; Spectra-Physics, Inc.) was combined with the OPO beam using a dichroic filter.

### Image Processing

Image processing was done using ImageJ (Schneider et al., 2012) and MATLAB (MathWorks, Inc., Natick, MA, USA). Mosaics were created using MosaicJ (Thevenaz and Unser, 2007). For 3D visualization, the contrast of the z-stack was adjusted (Capek et al., 2006) and rendered using Amira (Thermo Scientific). Axon tracing was performed by single-particle tracking (Crocker and Grier, 1996; Chenouard et al., 2014) and semiautomatic ridge detection (Meijering et al., 2004; Longair et al., 2011).

## Results

### THG Arises From Compact Myelin in the Cerebral Cortex

**Figure 1:**
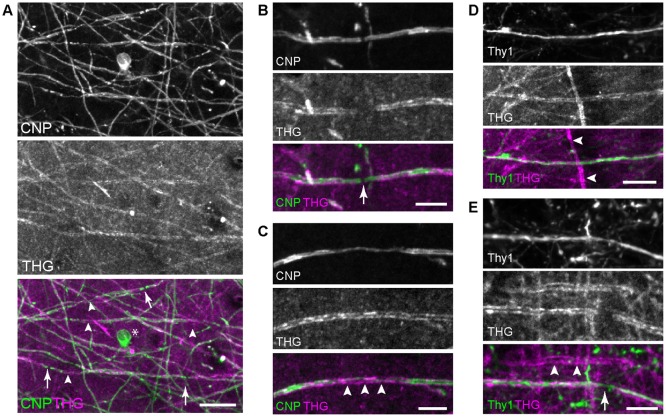
Imaging the whole brains of 2′,3′-cyclic nucleotide 3′ phosphodiesterase (CNP)-green fluorescent protein (GFP) and Thy1-yellow fluorescent protein (YFP) mice. **(A)** Co-registration of CNP-GFP and third-harmonic generation (THG), with the node of Ranvier (arrows) and compact myelin where CNP tapers (arrowheads). The cell body is also labeled by CNP-GFP (*). Scale bar, 20 μm. **(B,C)** A magnified view around the node and the compact myelin, respectively. Scale bars, 10 μm. **(D,E)** Myelinated Thy1+ cells with the node (arrow) showing axoplasmic Thy1 within the THG gap. Thy1- cells can be also myelinated (arrowheads). Scale bars, 20 μm.

First, THG was tested for visualizing myelin domains. Transgenic mice CNP-green fluorescent protein (GFP) and Thy1-YFP were employed, which expressed membrane-anchored GFP in myelinating cells under the CNP promoter (Deng et al., 2014) and YFP in the cytoplasm of neurons under the Thy1 promoter (Feng et al., 2000), respectively. From the brain from young adult mice, 2PEF and THG were acquired simultaneously for co-registration. We imaged both fixed and unfixed specimens from which comparable THG signals were obtained. Figure 1 depicts representative images of the visual cortex of the fixed whole brain (V1, layer 1). Substantial co-localization between CNP-GFP and THG confirmed that the origin of THG was primarily myelinated axons. Not all fibers expressing CNP-GFP appeared in the THG channel (Figure 1A), indicating a relatively lower sensitivity of THG. Approximately 50% of CNP+ fibers were visualized by THG; those that were THG+ had larger fiber diameters. Interestingly, THG was often complimentary to CNP-GFP; it was stronger in the compact myelin just as in Schwann cells (Lim et al., 2014), whereas CNP-GFP was enriched in non-compact intracellular domains (Deng et al., 2014) analogously to the native CNP protein itself (Peirce et al., 2006). Consequently, the cell body of the OL (asterisk, Figure 1A) and the paranode (arrow, Figure 1B) were visible by CNP-GFP while there was a chasm of THG at the node of Ranvier. THG revealed the full width of internodes, while the subcellular localization of CNP-GFP was often found within narrow strips along the internode (arrowheads, Figure 1C). The co-registration in the Thy1-YFP brains verified that cortical myelination was not specific to the cell type; both Thy1+ and Thy1- cells could be myelinated or unmyelinated (Figures 1D,E).

### The Relationship Between Cyto- and Myeloarchitecture

**Figure 2:**
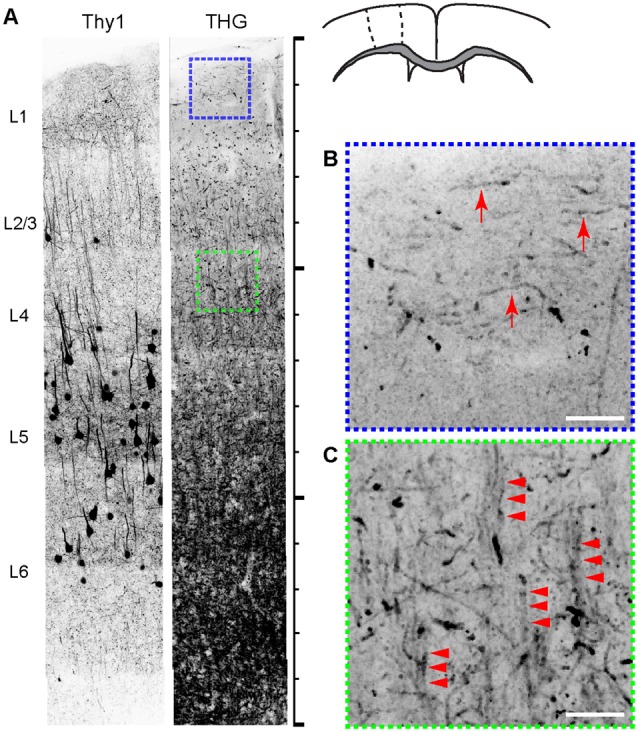
The relationship between cyto- and myeloarchitecture of the cerebral cortex by Thy1-YFP and THG, respectively (inverted contrasts). **(A)** The coronal section. Scale bar, 1,500 μm. **(B,C)** Magnified views of regions shown in **(A)**. THG images of layers 1 (blue) and 4 (green). Myelinated fibers tangential (arrows) and radial to the pia (arrowheads) appear in specific layers. Scale bar, 25 μm.

The intricate organization of neuronal and non-neuronal cells has a significant bearing on the physiological and cognitive functions of the cerebral cortex. We compared the cyto- and myeloarchitecture of the same brain by simultaneously acquired 2PEF and THG from Thy1-YFP mice. Figure 2 depicts a fixed coronal section of the primary motor cortex (M1). The density of myelinated fibers increased monotonically with depth. Furthermore, the structure of myelinated fibers, which was lattice-like, also varied with depth; the fibers in layer 1 were distinctly tangential to the pia (arrows, Figure 2B), i.e., the plexus of Exner, while those in layers 2/3 were predominantly radial. The radial fibers were diffuse in layers 2/3 but more bundled in layer 4 (arrowheads, Figure 2B). The overall morphological similarity to the human anatomy (Baillarger, 1840; Vogt, 1910; Vogt and Vogt, 1919; Hopf, 1967) affirmed the rodent as a viable model. However, the complete myeloarchitecture was difficult to achieve with the two-dimensional slices where the fibers perpendicular to the section are underrepresented. In particular, it was unclear whether there were dense tangential fibers in deeper cortical layers (>layer 4) resembling the bands of Baillarger in humans—the lack of columnar striations myelinated fibers similar to Thy1+ cell bodies (Figure 2A) could be a mere sectioning artifact. To address this, the morphology of the myelin network must be examined in a three-dimensional volume of the brain.

### The Depth of THG Imaging of Cortical Myelin in the Live Mouse Brain

**Figure 3:**
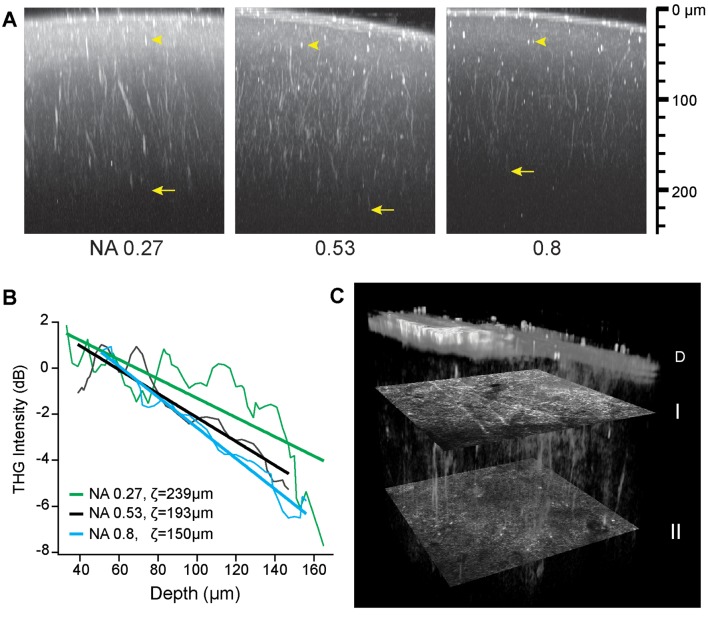
The depth of THG imaging of the intact mouse brain. **(A)** Characterizing the resolution (arrowheads) and depth (arrows) of *ex vivo* imaging. Maximum-intensity projection is shown for three effective NAs on the logarithmic scales. **(B)** Decreasing THG intensity with depth at a rate depending on numerical aperture (NA). The normalized THG intensity from radial myelinated fibers (average of *N* = 6) and the best fit to a linear model *log〈I/I*_0_〉 = (*z*_0_ − *z*)/ζ. **(C)** Cortical myelin imaged in a live animal. Volumetric rendering of a region (151 μm × 151 μm × 186 μm) shows distinct distribution of tangential and radial fibers at depths of 43 and 134 μm below the dura D (transverse sections I and II, respectively).

For volumetric imaging of the intact brain, first the depth of THGM was characterized. A major limiting factor is specimen-induced aberration, i.e., optical aberration arising from the turbidity of tissue (Hell et al., 1993; Booth et al., 2002; Ji et al., 2012), causing the quality of the laser focus to degrade with depth. To characterize the effect of the numerical aperture (NA) of the focusing excitation beam on the rate of degradation, we varied the NA by underfilling the back aperture of the objective lens with the beam diameter, thus maintaining the efficiency of signal collection. The average excitation power was constant at the sample. The effective NA can be defined for an incident Gaussian beam truncated by the entrance pupil (Dickson, 1970).

$${\text{NA}}_{\text{eff}}{\text{=NA}}_{\text{obj}}\text{×}\frac{\text{Gaussian beam diameter (FWHM)}}{\text{2×Entrance pupil diameter}}$$

where NA_obj_ is the nominal NA of the objective lens and the beam diameter is the full width at the half maximum (FWHM). Figure 3 shows the three maximum-intensity projections of the axial sections of a fixed whole mouse brain acquired at the effective NAs of 0.27, 0.53, and 0.8. The maximum depth of approximately 200 μm was achieved at the intermediate NA of 0.53 (arrows, Figure 3) due to a tradeoff between the power density and specimen-induced aberration. At the effective NA of 0.27, the laser focus was larger, as verified by the relative size of punctate features (arrowheads, Figure 3), so the depth range was limited by the low power density. However at the effective NA of 0.8, despite the tighter focus near the pia, the sample-induced aberration became dominant with depth resulting in the reduced range. Having determined the optimal effective NA of ~0.53, we performed intravital THGM through an optical cranial window and a similar depth of ~200 μm was achieved (Figure 3C). Remarkably, THG visualized myelinated fibers of all orientations unraveling the depth-dependent distribution of myelin (I and II, Figure 3C).

### Quantification of the Depth-Dependent Distribution of Myelinated Fibers

**Figure 4:**
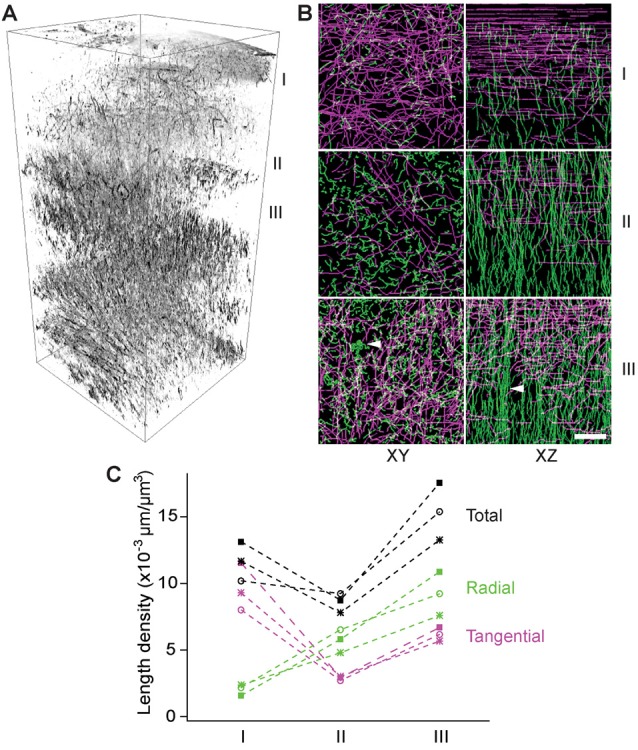
The depth-dependent distribution of tangential and radial fibers. **(A)** Volumetric rendering of THG stacks in the range of 0–800 μm of the cortex. I, II, and III: 0–100 μm, 200–300 μm, and 300–400 μm below the dura, respectively (inverted contrasts). **(B)** The transverse (XY) and axial (XZ) projections of traced THG-positive axons exhibiting a lattice structure and fiber bundles (arrowheads) in deeper layers. Scale bar, 20 μm. **(C)** The length density of traced THG-positive fibers (*N* = 3).

To examine the layer-specific orientations of myelinated fibers, we made transverse slices of a fixed brain with a thickness of 100 μm (i.e., a half of the imaging depth) for THG imaging analogous to the previous serial-section tomography (Denk and Horstmann, 2004; Micheva and Smith, 2007; Ragan et al., 2012). Z-stacks were acquired in the first eight slices covering a range of 0–800 μm deep and a volumetric rendering was created (Figure 4A). The fibers of all orientations could be visualized. The myelin network exhibited a lattice structure, as previously seen by IHC, where the fibers could be classified unambiguously as either radial or tangential. There were significant depth-dependent variations in terms of the total and relative abundance of the radial or tangential fibers. To analyze this quantitatively, a 3D volume of 100 μm × 100 μm × 100 μm was selected at three different depths in the cerebral cortex, i.e., 0–100 μm, 200–300 μm, and 300–400 μm (I, II, and III in Figure 4, respectively) and the myelinated axons within each volume were traced semi-automatically. The axial projection of traced axons (XZ, Figure 4B) replicated the results of Figures 2B,C, i.e., that the density of radial fibers increased monotonically with depth and some fibers merged to form bundles in deeper cortical layers (arrowhead, Figure 4B). Furthermore, distinct bands of tangential fibers could be observed in deeper layers (volume III, Figures 4B,C). The total length density of myelinated fibers in the range of 300–400 μm (layers 2/3) that were visualized by THG was approximately 0.015 μm/μm^3^, which corresponds to a volume fraction of ~17%. Previously, the length density has been determined to be ~0.040 μm/μm^3^ for the human and chimpanzee brains at an unspecified depth (Miller et al., 2012). For mice, we obtained a similar density by CNP-GFP, but a smaller value within an order of magnitude by THG because of a bias against thinner axons.

### Optical Morphometry of the Cortical Myelin Sheath

**Figure 5:**
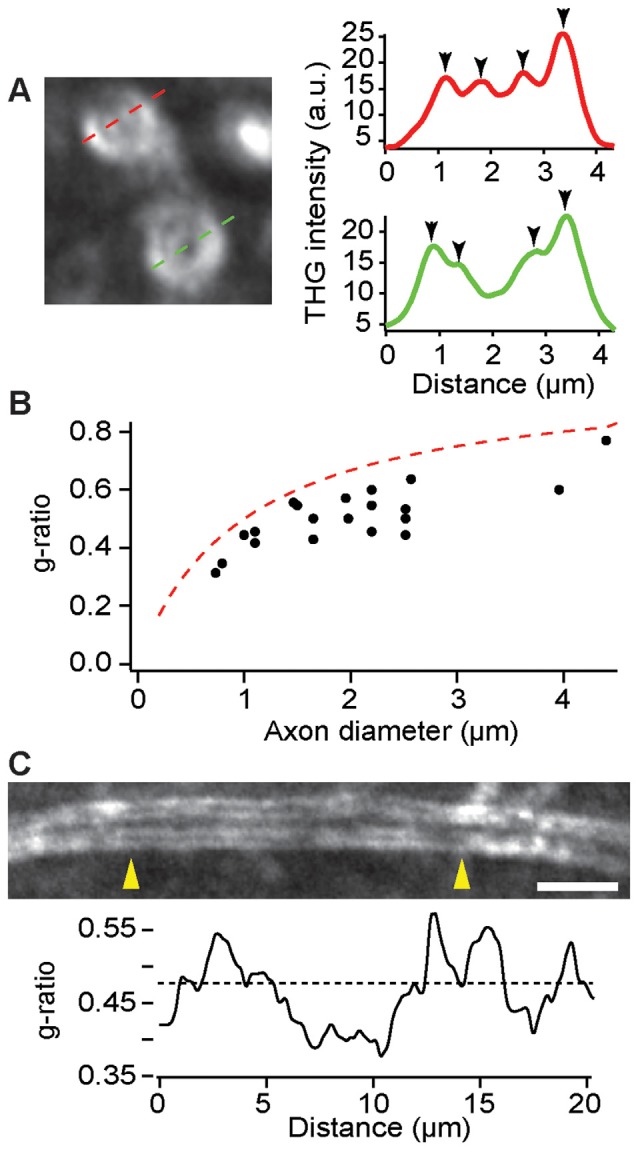
Evaluation of the g-ratio in the deeper cortex by THG. **(A)** The cross sections of myelinated axons (left) and the corresponding intensity profiles (right). The position of adaxonal and abaxonal membranes can be precisely determined (arrowheads, right). **(B)** The g-ratio vs. the axon diameter for a total of 20 axons in layer 6 of the primary motor cortex (M1) of C57BL/6 mice (*N* = 5). The dashed line is for the case of a 0.5-μm myelin sheath. **(C)** A single internode at a depth of approximately 500 μm exhibiting variations of the g-ratio across the length between arrowheads (yellow). The dashed line denotes the average g-ratio. Scale bar, 5 μm.

As an important indicator of the conduction velocity, the ratio of axon to fiber diameter, namely the g-ratio, could be modulated during learning and pathology. On account of the sensitivity to the boundaries between lipids and aqueous medium, THGM is capable of measuring the biometric precisely (Lim et al., 2014), so that remodeling of myelin can be studied quantitatively in fresh tissue. The precision of THG-based morphometry is inevitably limited by the optical resolution and we found that most myelin sheaths in the cerebral cortex were too thin to be resolved. Nonetheless, the adaxonal and abaxonal membranes could be discriminated for large caliber axons in the deeper cortex (>500 μm) of a fixed, dissected brain (Figure 5A). The g-ratio in a region within layer 6 of the M1 confirmed the typical values of the g-ratio in the CNS as well as the limitation of THG morphology (Figure 5B). There were also substantial variations of the g-ratio along single internode (Figure 5C), similar to the PNS (Lim et al., 2014).

## Discussion

We have demonstrated THG for imaging myeloarchitecture in the living cerebral cortex. As a label-free modality, it is applicable to most vertebrates, i.e., not limited to fluorescently-labeled transgenic mice, thus simplifying experimental designs for studying neurological disorders in models where OL markers, such as CNP and myelin-associated glycoprotein (MAG), are dysregulated (Davis and Haroutunian, 2003; Barley et al., 2009). The axon’s decision to myelinate or demyelinate has long-range consequences affecting not only proximal but also distal regions in the cerebrum. Tracking myelinated fibers within living brains is invaluable for studying the role of dynamic remodeling during learning or disease. Furthermore, a comprehensive atlas of the myelin network in 3D mouse brain acquired by THG tomography will be a substantial resource for neuroscience research.

Because the THG signal arises from interfaces of all orientations (Cheng and Xie, 2002), both tangential and radial fibers could be acquired without a directional bias. It is therefore distinguished from reflectance-based modalities, e.g., spectral confocal reflectance microscopy (SCoRe; Schain et al., 2014) and optical coherence tomography (OCT; Ben Arous et al., 2011; Leahy et al., 2013), which visualizes only a subpopulation of the fibers that are perpendicular to the optic axis. Moreover, the sensitivity to the lipid-aqueous boundary discriminates THG from another myelin contrast, i.e., coherent anti-Stokes Raman scattering (CARS; Wang et al., 2005; Fu et al., 2008; Imitola et al., 2011), which originates from all lipids within a 3D volume. The surface-dependence of THG is more ideal for morphometry of the myelin sheath because the position of the adaxonal and abaxonal membranes can be determined precisely, conceivably even beyond the optical resolution (Bobroff, 1986).

Future developments are anticipated for improving intravital THG imaging of cortical myelin, especially to extend the depth range. We achieved an imaging depth of ~200 μm into the brain *in vivo* using an OPO as a light source. The use of a single short-pulse laser is a significant merit, as opposed to two synchronized pulsed lasers required for CARS in which chromatic aberration imposes an additional factor limiting the depth range. However, while substantially better than that of confocal microscopy including SCoRe (~50 μm), the depth of intravital THG imaging is still less than that of typical 2PEF microscopy (>500 μm). For the reconstruction of the whole cortex *in vivo*, a deeper range is desirable. The result of Figure 3 suggests that the methods of wavefront correction (Neil et al., 2000; Ji et al., 2012) can be effective for deeper THG imaging beyond 200 μm. Employing a higher average power is also feasible but at an elevated risk of photodamage from excessive thermal energy. Alternatively, a special light source, as previously demonstrated for THG (Farrar et al., 2011; Tokarz et al., 2017) and 3PEF microscopy (Horton et al., 2013), could significantly increase the range of THG imaging of gray-matter myelin in the cerebrum.

## Data Availability

All datasets generated for this study are included in the manuscript.

## Ethics Statement

This study was carried out in accordance with recommendations of the Hunter College Institutional Animal Care and Use Committee (IACUC). The protocol was approved by the Hunter College IACUC.

## Author Contributions

MR performed the experiments, analyzed the data, and wrote the manuscript. HL designed experiments, analyzed the data, and wrote the manuscript.

## Conflict of Interest Statement

The authors declare that the research was conducted in the absence of any commercial or financial relationships that could be construed as a potential conflict of interest.
